# Study design of VGOAL-J: an observational, prospective cohort study to assess effectiveness of vortioxetine on goal achievement and work productivity in patients with MDD in Japan

**DOI:** 10.1192/j.eurpsy.2022.1411

**Published:** 2022-09-01

**Authors:** K. Watanabe, Y. Moriguchi, H. Ren

**Affiliations:** 1 Kyorin University School of Medicine, Department Of Neuropsychiatry, Tokyo, Japan; 2 Lundbeck Japan, Medical Affairs, Tokyo, Japan; 3 H.Lundbeck A/S, Medical Affairs, Valby, Denmark

**Keywords:** real world evidence, vortioxetine, Depression, patient centricity

## Abstract

**Introduction:**

Goal attainment scaling (GAS) is a method to assess the patient experience of whether a treatment is successful and capture outcomes across a diverse range of goal areas. However, this approach has not yet been used in assessing the treatment of Major Depressive Disorder (MDD) in Japan. GAS was first developed by Kiresuk and Sherman in the 1968, it is increasingly recognised as a sensitive method for recording patient-centred outcomes throughout the course of treatment.

**Objectives:**

To demonstrate the effectiveness of vortioxetine on patient’s goal achievement and depressive symptoms, emotional, cognitive, overall function and quality of life.

**Methods:**

VGOAL-J is a prospective, multi-center, observational cohort study of outpatients initiating vortioxetine treatment for MDD in Japan. Patients with a diagnosis of MDD according to DSM-5 who are 18 to 65 years will be enrolled from 20 sites in Japan and followed for 24 weeks. A total number of 120 patients is planned for enrolment. Primary outcome measures are GAS-D, WPAI, secondary outcome measures include Montgomery – Åsberg Depression Rating Scale (MADRS), Sheehan Disability Scale (SDS), Perceived Deficits Questionnaire-Depression 5-item (PDQ-D-5), Oxford Depression Questionnaire (ODQ), EuroQol-5 Dimension (EQ-5D). Safety will be also assessed with Adverse Events collected during the study.

**Results:**

The results will be disseminated in late 2022 and provide new insights on GAS-D as an effective strategy to assess MDD treatment in Japan.

**Conclusions:**

We expect to observe patients treated with vortioxetine achieving their treatment goals as assessed by GAS-D and improvements on patient- and clinician-reported measures in real-world settings.

**Disclosure:**

Prof. K. Watanabe reports consultancies undertaken for Eli Lilly, Otsuka Pharmaceutical, Sumitomo Dainippon Pharma, Taisho Toyama Pharmaceutical, and Takeda Pharmaceutical, honoraria received from Daiichi Sankyo, Eisai, Eli Lilly, GlaxoSmithKline, J

